# Use of plasma mitochondrial DNA levels for determining disease severity and prognosis in pediatric sepsis: a case control study

**DOI:** 10.1186/s12887-018-1239-z

**Published:** 2018-08-09

**Authors:** Hai peng Yan, Miao Li, Xiu lan Lu, Yi min Zhu, Wen-xian Ou-yang, Zheng hui Xiao, Jun Qiu, Shuang jie Li

**Affiliations:** 1 Department of Pediatric Intensive Care Unit (PICU), Hunan Children’s Hospital, Changsha, China; 2 Hunan Provincial People’s Hospital, the first affiliated hospital of Hunan normal University, Changsha, 410007 People’s Republic of China; 3 Department of Section of Liver Disease, Hunan Children’s Hospital, 86# Ziyuan Road, Changsha, 410007 China

**Keywords:** Mitochondrial DNA, Sepsis, Multiorgan failure, Prognosis, Biomarker

## Abstract

**Background:**

The mortality rate due to severe sepsis is approximately 30–60%. Sepsis readily progresses to septic shock and multiple organ dysfunction, representing a significant problem in the pediatric intensive care unit (PICU). The aim of this study was to explore the value of plasma mitochondrial DNA (mtDNA) for early diagnosis and prognosis in children with sepsis.

**Methods:**

A total of 123 children with sepsis who were hospitalized in the Hunan Children’s Hospital PICU from July 2013 to December 2014 were divided into the general sepsis group (*n* = 70) and severe sepsis group (*n* = 53) based on diagnostic standards. An additional 30 children with non-sepsis infection and 30 healthy children were randomly selected as a control group. Patients’ plasma was collected during admission to the PICU. A pediatric critical illness score (PCIS) was also calculated. The plasma mtDNA level was examined using real-time polymerase chain reaction technology, and other parameters including routine laboratory values; blood lactate, procalcitonin (PCT), and C-reactive protein (CRP) levels; and data on survival were collected and compared among the groups.

**Results:**

The plasma mtDNA level in the sepsis group than that in the non-sepsis infection and healthy groups. The plasma mtDNA level was significantly higher in the severe sepsis than in the general sepsis group (*p* < 0.001). A lower PCIS was associated with a higher plasma mtDNA level (*p* < 0.001). A higher number of organs with dysfunction was associated with higher plasma mtDNA levels (*p* < 0.001). Plasma mtDNA levels were higher among patients with elevated alanine aminotransferase, aspartate aminotransferase, blood urea nitrogen, creatinine, lactate dehydrogenase, creatine kinase, myoglobin, creatine kinase MB, and troponin than in those with values within the normal range. The mtDNA level was higher among non-survivors than among survivors, and this difference was significant. mtDNA showed a prognostic prediction value similar to that of lactate, PCT, and CRP.

**Conclusions:**

Plasma mtDNA levels may be a suitable biomarker for diagnosis and prognosis in children with sepsis.

## Background

Sepsis is a systemic inflammatory response syndrome caused by infection. Its mortality and incidence rate are extremely high, with the mortality rate for severe sepsis reported to be 30–60% [1]. Sepsis can easily progress to septic shock and multiple organ dysfunction, which has become a significant problem in the pediatric intensive care unit (PICU).

Early diagnosis of severe sepsis using reliable biomarkers is essential for reducing mortality. So far, more than 170 biomarkers have been developed for predicting morbidity and mortality in the critical care setting [2, 3], including lactate, procalcitonin (PCT), and C-reactive protein (CRP); in addition, some scoring systems are also used for prognostic prediction. Plasma-free DNA can be defined as DNA fragments detectable in extracellular fluid that are of two types: mitochondrial DNA (mtDNA) and nuclear DNA; plasma-free DNA can be released into the blood from apoptotic and necrotic cells [4, 5]. Circulating mtDNA has a characteristic short length, simple structure, and high copy number and has been proven to have a damage-associated molecular pattern reflecting cellular injury in trauma and microbial infection [6, 7]. Moreover, mtDNA can drive molecular processes, leading to inflammatory responses and organ injuries [8, 9]. Thus, mtDNA has received extensive attention in the study of many diseases including cancer [10], ischemic stroke [11], prediabetes [12], and trauma [13]. Several studies have reported that plasma mtDNA levels show abnormal elevation in cases of sepsis in adults and that they are useful for determining the severity of sepsis [14–17]. mtDNA is also reported to predict mortality with higher specificity and sensitivity than PCT, CRP, and other indicators [16, 18–20]. However, only a few studies focused on the use of mtDNA to predict the prognosis in children with sepsis.

Hence, we hypothesized that plasma mtDNA levels could be associated with disease severity and could also predict the prognosis in children with sepsis. The objective of this study was to investigate whether circulating cell-free mtDNA levels can be a useful biomarker for sepsis in the pediatric population.

## Methods

### Patients and groups

The study was approved by the Clinical Research Ethics Committee of the Hunan Children’s Hospital Center. Informed written consent was also obtained from a legal guardian of each child involved in our study before data collection. Between July 2013 and December 2014, patients with pediatric sepsis and infected patients without sepsis who were admitted to the PICU of Hunan Children’s Hospital (University of South China, Changsha, China) were enrolled in this study. The diagnostic criteria for sepsis were based on the “Surviving Sepsis Campaign: International Guidelines for Management of Severe Sepsis and Septic Shock, 2012” [21]. Patients with underlying diseases, such as hematological disease, congenital heart disease, metabolic diseases, hepatopathy, nephropathy, and malignant tumor, were excluded.

Furthermore, patients were divided into the general sepsis group, severe sepsis group, non-sepsis infection group according to the diagnostic criteria. Moreover, children were also randomly selected for healthy physical examination in our hospital and classified under the normal control group. The pediatric critical illness scores (PCIS) were calculated using the formula previously published in literature [22]. Moreover, patients were divided according to their PCIS scores: > 90 group, 81 ~ 90 group, 71–80 group, and ≤ 70 group. Subsequently, patients were divided into the survival and non-survival group according to the hospital outcome. Patients were also classified into different groups according to the severity of organ damage. Damage to the liver, heart, and kidney were evaluated according to their representative laboratory enzyme levels.

### PCT and lactate measurement

Blood samples were collected during the first hour of the hospital admission. Blood chemistry and blood gas analysis; examination of PCT, lactate, and CRP levels; and blood, sputum, urine, and stool cultures were performed at the Laboratory Department at Hunan Children’s Hospital Center.

### Methodology of DNA isolation and quantitative polymerase chain reaction (qPCR)

**Fig. 1:**
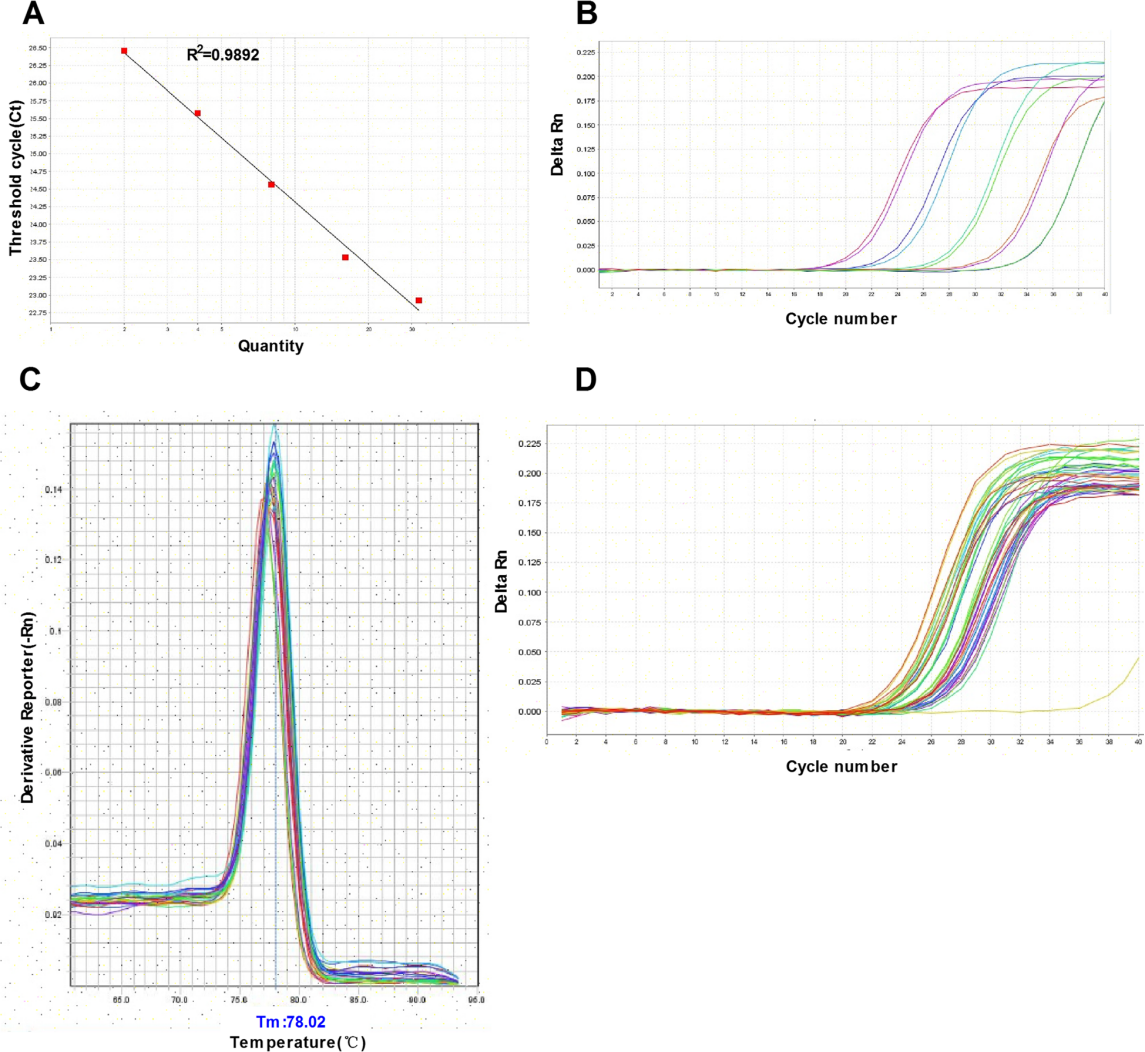
Representative standard curve, amplification plot, and dissociation curve of patients’ samples. **a** The assay was linear for DNA copy numbers (R^2^ = 0.9892). **b** The amplification plot showed no regular amplification for the standard diluents. **c** The dissociation curve showed a single melting temperature of specific products generated with a standard template. These dissociation curves indicated that the reactions were free of primer-dimer or any other spurious products. **d** Similar amplification plots were observed after quantitative polymerase chain reaction using patients’ samples. The amplification curves generated from human samples were paralleled with the curves from standards and were in the range of amplification plots for standard diluents

The methodology of DNA isolation and qPCR was strictly based on the manufacturer’s instructions. In brief, plasma used in this study was collected using ethylenediaminetetraacetic acid-coated blood collection tubes and centrifuged at 700 g at 4 °C for 5 min. DNA from plasma was isolated using the QIAamp DNA blood Mini Kit (Qiagen, Germany). Then, real-time qPCR was performed to quantify the mtDNA. Initially, primers were designed by referring to a previous publication [16], and the sequences were as follows: mtDNA, forward 5’-CAC AGA AGC TGC CAT CAA GTA-3’; reverse 5’-CCG GAG AGT ATA TTG TTG AAG AG-3’. The specificity of mtDNA primers using a Genbank search was examined, and mtDNA concentration was determined by a spectrophotometer. A representative standard curve, dissociation curve, and amplification plot are shown in Fig. 1.

### Statistical analysis

Statistical analysis was performed using the SPSS for Windows version 18.0 (IBM SPSS Inc., Armonk, NY, USA). All data were tested for normality. Data with a normal distribution are reported as mean ± standard deviation (χ ± s), whereas data with a non-normal distribution are reported as median and interquartile range. Lactate levels were compared between the two severity groups and between the two prognosis groups using t-test. Lactate levels were compared between the four PCIS groups, and the three groups were defined based on the degree of organ damage using the ANOVA test. The mtDNA level was compared between the four groups using the Kruskal-Wallis H test. mtDNA and PCT levels were compared between the four PCIS groups using the Kruskal-Wallis H test. mtDNA and PCT levels were compared between the three groups defined based on the degree of organ damage using the Kruskal-Wallis H test. The Mann-Whitney U test was used to compare mtDNA and PCT levels between the two severity groups and between the two prognosis groups. The Mann-Whitney U test was used to compare mtDNA levels between the two groups defined based on the degree of organ damage. A Spearman’s rank correlation test was used to evaluate the correlation between mtDNA level and PCT level, lactate level, and PCIS. The discrimination power of mtDNA, PCT, and lactate levels for predicting mortality was determined with receiver operating characteristic curve analysis. Differences were considered significant if the *p* value was < 0.05.

## Results

### Baseline characteristics

Patients were divided into the general sepsis group (*n* = 70), severe sepsis group (*n* = 53), 30 children with non-sepsis infection, and 30 healthy children as a control group. Table 1 reports the baseline characteristics of patients included in these cohorts. The mean age ranged of patients in all four groups was 1–2 years. Patients were also predominately male. Age and sex distribution was similar between the healthy group and the general sepsis group, between the healthy group and the non-sepsis infection group, and between the general sepsis group and the severe sepsis group (*p* > 0.05). Of the 123 patients with sepsis, 108 (87.81%) had a clear primary site of infection, including 70 cases of lung infection (56.91%), 21 cases of intracranial infection (17.07%), 12 cases of gastrointestinal infection (9.76%), and 5 cases of bloodstream infection (4.07%). The remaining 15 cases (12.19%) did not have a clear site of infection. In terms of pathogens, 52 cases were bacterial infection (42.23%), 11 were viral infection (8.93%), 6 were mycoplasma infection (4.88%), and 3 were fungal infection (2.44%). The remaining 51 cases have unclear etiology (41.46%).

**Table 1:** Baseline characteristics of patients

		Number (Percent) or Median (Interquartile Range)			
Category	Variable	Normal *n* = 30	General sepsis n = 70	Severe sepsis n = 53	Non-sepsis infection n = 30
Demographic parameters	Age (years)	2 (1 month~ 12)	1 (1 month~ 13)	2 (1 month~ 12)	1 (1 month~ 13)
	Gender (male)	18 (60%)	48 (68.57%)	29 (54.72%)	17 (56.67%)

### mtDNA levels in the four groups and variation according to sepsis severity

Table 2 presents the mtDNA levels in the general sepsis, severe sepsis, non-sepsis infection, and healthy groups. Moreover, the plasma mtDNA level in the general sepsis group was higher than that in the healthy group (667.35 vs 278.14, *p* < 0.01) and the non-sepsis infection group (667.35 vs 433.54, *p* < 0.05). In addition, the plasma mtDNA levels in the severe sepsis group were higher than those in the general sepsis group (1502.77 vs 667.35, *p* < 0.001).

**Table 2:** Correlation between mtDNA and different disease, PCIS score, organs damage and fatality

		mt-DNA				PCT			Lactate		
		Value (pg/ml)		Text Value	*p*	Value (ng/ml)	Text Value	*p*	Value (mmol/l)	Text Value	*p*
Disease	Normal	30	278.14 (185.14–351.56)		0.01^a^**						
	General sepsis	70	667.35 (412.58–893.71)			1.05 (0.17~ 3.41)	−6.58	0.000^d^***	1.1 ± 2.15	− 3.887	0.000^e^***
	Severe sepsis	53	1502.77 (1166.01–2221.88)	110.20	0.000^b^***	26.8 (4.51~ 174.33)			2.7 ± 3.45		
	Non-sepsis infection	30	433.54 (243.41–938.08)		0.05^c^*						
PCIS score	>90	34	647.20 (410.73~ 989.46)	24.33	0.000***	1.23 (0.17–6.35)	23.57	0.000***	1.49 ± 1.19	9.19	0.000***
	81~ 90	47	888.47 (474.645~ 1379.51)			2.51 (0.21–16.39)			1.88 ± 1.71		
	71~ 80	36	1184.82 (905.27~ 1997.40)			7.64 (1.36–137.51)			4.53 ± 3.97		
	≤70	7	1997.40 (1159.18~ 2601.67)			171.36 (12.97–200.00)			3.57 ± 4.54		
amount of organs damage	0	37	474.65 (346.14–876.79)	63.30	0.000***	0.35 (0.09–3.13)	38.40	0.000***	1.71 ± 1.52	7.081	0.001
	1	31	821.83 (522.42–1035.84)			2.51 (0.41–4.41)			1.84 ± 2.62		
	≧2	55	1454.78 (1141.13–2195.98)			12.97 (3.95–151.74)			3.72 ± 3.42		
ClinicalOut-come	Survival	97	865.10 (457.36–1277.47)	−3.534	0.000***	2.51 (0.28–11.04)	−3.642	0.000***	1.71 ± 1.52	3.7^f^	0.000***
	Non-survival	26	1269.89 (955.25–2063.11)			37.16 (3.35–141.87)			1.84 ± 2.62		

^a^means the mt-DNA value were compared between general sepsis group and normal groups

^b^means the mt-DNA value were compared between general sepsis group and Severe sepsis groups

^c^means the mt-DNA value were compared between general sepsis group and Non-sepsis infection groups

^d^means the PCT value were compared between general sepsis group and Severe sepsis groups

^e^means the lactate value were compared between general sepsis group and Severe sepsis groups

^f^means the Lactic value between survival group and non-survival groups were compared using t test

* means P<0.05. ** means P<0.01, *** means P<0.001

### Correlation between mtDNA levels and PCIS or organ damage

We classified patients into four groups according to PCIS assessment (> 90, 81–90, 71–80, and ≤ 70). As the PCIS decreased, the mtDNA level increased (*p* < 0.001; Table 2).

When patients were classified into three groups according to the degree of organ damage (0, 1, and ≥ 2), as the amount of organ damage increased, the mtDNA level also increased (*p* < 0.000; Table 2). Our results also showed that mtDNA levels were significantly higher in patients whose organ damage was more severe (Table 3).

**Table 3:** The mtDNA level in different organs damage enzymology indexes groups

Organs damage indexes	Group	Cases	mtDNA level	Z	*p* value
ALT (IU/L)	≦40	61	774.34(423.62–1100.88)	−4.512	0.000
	> 40	62	1196.43(829.45–2018.92)		
AST (IU/L)	≦40	42	678.21(412.59–1060.09)	−3.899	0.000
	> 40	81	1141.13(775.61–1757.91)		
LDH (IU/L)	≦450	66	689.05(425.76–1033.44)	−5.050	0.000
	> 450	57	1264.85(914.34–2002.52)		
CK (IU/L)	≦140	57	821.83(444.95–1197.90)	−2.855	0.004
	> 140	66	1166.01(790.16–1588.23)		
Mb (IU/L)	≦90	78	823.83(257.06–1298.33)	−3.214	0.001
	> 90	45	1180.13(889.31–1798.05)		
CK-MB (IU/L)	≦24	73	812.97(435.46–1176.82)	−4.305	0.000
	> 24	50	1196.43(897.29–1999.96)		
cTa	< 0.01	78	823.83(257.07–1298.33)	−3.214	0.001
	≧0.01	45	1180.13(889.31–1798.05)		
BUN (IU/L)	≦8.2	89	865.10(457.36–1284.09)	−3.26	0.001
	> 8.2	34	1182.48(910.89–2123.57)		
Cr (umol/L)	≦120	107	891.99(475.89–1379.99)	−2.71	0.007
	> 120	16	1247.94(982.85–1897.73)		

### Correlation between mtDNA levels and mortality

As shown in Table 2, the mtDNA level was higher in the non-survival group than in the survival group (1269.89 vs 865.10, *p* < 0.001).

### Comparison of mtDNA with lactate, PCT, and CRP with respect to prognostic value

In the severe sepsis group, PCT and lactate levels were all higher than those in the general sepsis group, similarly with the mtDNA level (*p* < 0.001; Table 2).

When patients were classified into four groups according to PCIS assessment, classified into three groups according to the amount of organ damage, and classified into non-survival and survival groups, PCT and lactate levels showed trends similar to those of the mtDNA level, with significant group differences; however, this was not observed for the lactate value when the PCIS was less than 70 (Table 2).

**Fig. 2:**
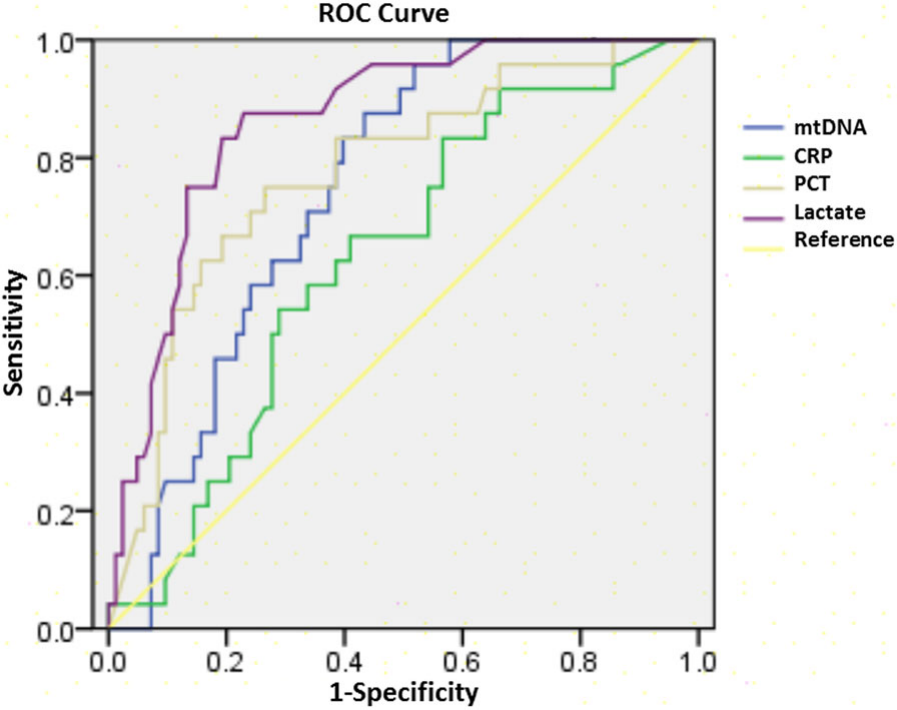
The diagnostic accuracy of different biomarkers in predicting mortality. The receiver operating characteristic curve shows the diagnostic accuracy of mitochondrial DNA (blue), C-reactive protein (green), procalcitonin (gray), and lactate (purple). The yellow line is the reference

To further assess the value of mtDNA as a prognosis biomarker, we compared the ability of mtDNA, lactate, PCT, and CRP levels to differentiate survival from non-survival groups. The optimal cut-off values (sensitivity and specificity) were 890.43 (88.5, 53.6%) for mtDNA, 2.15 (88.5, 77.4%) for lactate, 6.88 (73.1, 71.3%) for PCT, and 19.95 (66.7, 60.2%) for CRP (Table 4). The area under the curve (AUC) was 0.726 for mtDNA, 0.864 for lactate, 0.776 for PCT, and 0.629 for CRP (Fig. 2).

**Table 4:** Diagnostic Efficacy of mtDNA level, lactate, PCT and CRP in differentiation between the survival and non-survival groups

	AUC	Cut-off	Sensitivity (%)	Specificity (%)	*p* value
mtDNA (pg/ml)	0.726	890.43	88.5	53.6	0.000
Lactate (mmol/l)	0.864	2.15	88.5	77.4	0.000
PCT (ng/ml)	0.776	6.88	73.1	71.3	0.000
CRP (mg/l)	0.629	19.95	66.7	60.2	0.054

## Discussion

In the present study, we investigated plasma mtDNA levels in pediatric patients with sepsis. Our primary results are as follows: (1) the plasma mtDNA levels were significantly higher in the severe sepsis group than those in the general sepsis, non-sepsis infection, and healthy groups; (2) the mtDNA level is clearly associated with disease severity and mortality; (3) mtDNA levels showed a prognostic prediction value similar to that of PCT and lactate levels.

In 2016, Valentina first reported on plasma mtDNA levels in the pediatric population, including a small sample of 28 patients with sepsis, 20 healthy controls, and 10 critically ill non-septic patients. In the current study, we expanded the sample size to include 123 children with sepsis, 30 children with non-sepsis infection, and 30 healthy children. Our findings were consistent with the results reported by Valentina, demonstrating that plasma mtDNA concentrations were significantly elevated in patients with sepsis compared to those without sepsis and the control group [23]. We further revealed that higher mtDNA levels correlated significantly with organ dysfunction, by analyzing organ damage based on enzyme levels and comparing mtDNA levels to patients without organ dysfunction. A separate study in adult sepsis showed that mtDNA levels increased on day 1 and remained constant until day 5 in patients diagnosed with sepsis [24]. The mechanisms for mtDNA release involve two steps: cytosolic followed by extracellular actions. In the former, mitochondrial permeability transition pores in the inner mitochondrial membrane play a critical role. Then, cellular stress and necrosis are the primary factors leading to mtDNA release [25]; imbalanced production of reactive oxygen species and defects in energy production also play roles in producing increased mtDNA levels [26].

Our study demonstrated that the mtDNA level is clearly associated with disease severity and mortality. Several adult studies focused on the mechanism of mtDNA-induced organ dysfunction [27]. It was reported that toll-like receptor 9 (TLR9) is involved in multiple organ failure, including acute kidney injury (AKI) and sepsis-induced cardiac inflammation. Moreover, mtDNA can lead to upregulation of plasma IL-12, splenic apoptosis, and mitochondrial injury in TLR9-associated septic AKI [28]. Thus, researchers have focused on TLR4/MEK/ERK/TNF-α signaling, with the aim of developing a method to prevent mitochondrial dysfunction and AKI induced by sepsis [29].

Furthermore, our data showed that plasma mtDNA levels could predict prognosis. The plasma mtDNA level in the non-survival group was higher than that in the survival group. A study of patients with severe sepsis in the emergency room showed that plasma mtDNA levels were an independent predictor of fatality and that an increase of 1 ng/mL in the mtDNA level correlated with an increase of 0.7% in the rate of fatality [16]. A previous study in a medical ICU showed that patients with an elevated mtDNA level (≥ 3200 copies/μl plasma) had increased odds of dying within 28 days of ICU admission [30]. Other studies reported that mtDNA haplogroups JT and HV showed improved 30-day survival compared to non-JT and IWX haplogroups [31–33].

Notably, the plasma mtDNA level did not have the highest AUC value for predicting mortality compared with PCT and lactate. We cannot ignore the fact that lactate is closely related to tissue oxygen supply and that the level of lactate changes rapidly. Additionally, PCT is often used as an index of bacterial infection. Sepsis can also develop secondary to other types of infection, including viral and fungal diseases. For CRP levels, multiple studies have reported that CRP lacks the specificity to discriminate between bacterial, viral, and noninfectious inflammatory conditions. The CRP level is elevated in many inflammatory conditions and in rheumatologic, gastroenterologic, and cardiologic diseases [34]. Thus, we suggest that it is better to combine mtDNA and lactate levels in the context of a full clinical examination, in addition to evaluating the presence of other signs and symptoms, to assess the severity of sepsis in children.

This study has some limitations. A serial measurement of mtDNA, PCT, and lactate levels during sepsis was not performed. Further large-scale cohort and multi-center pediatric studies are warranted to confirm our results and to elucidate the mechanisms underlying the correlation between mtDNA and sepsis.

## Conclusions

In summary, plasma mtDNA might be a suitable biomarker for diagnostic and prognostic use in children with sepsis. Further investigations on the mechanism of mtDNA liberation and its function in the extracellular milieu might lead to new strategies for preventing septic shock and multiple organ dysfunction syndrome.

## Abbreviations

AKI: Acute kidney injury
AUC: Area under the curve
CRP: C-reactive protein
mtDNA: Plasma mitochondrial DNA
PCIS: Pediatric critical illness score
PCT: Procalcitonin
PICU: Pediatric intensive care unit
qPCR: Quantitative polymerase chain reaction
TLR9: Toll-like receptor 9
